# Supplementary material for: Causal interplay between lactose intolerance and gut microbiota: a combined bidirectional Mendelian randomization and in vivo validation study
Source: Front Nutr. 2026 Jun 1;13:1803337. doi: 10.3389/fnut.2026.1803337 (PMC13265576; doi:10.3389/fnut.2026.1803337)
Supplement: Supplementary file 1 [file Data_Sheet_1.zip › supplementary materials/Forward/forest plot/ebi-a-GCST90027642.finngen_R12_E4_LACTONAS.pdf]

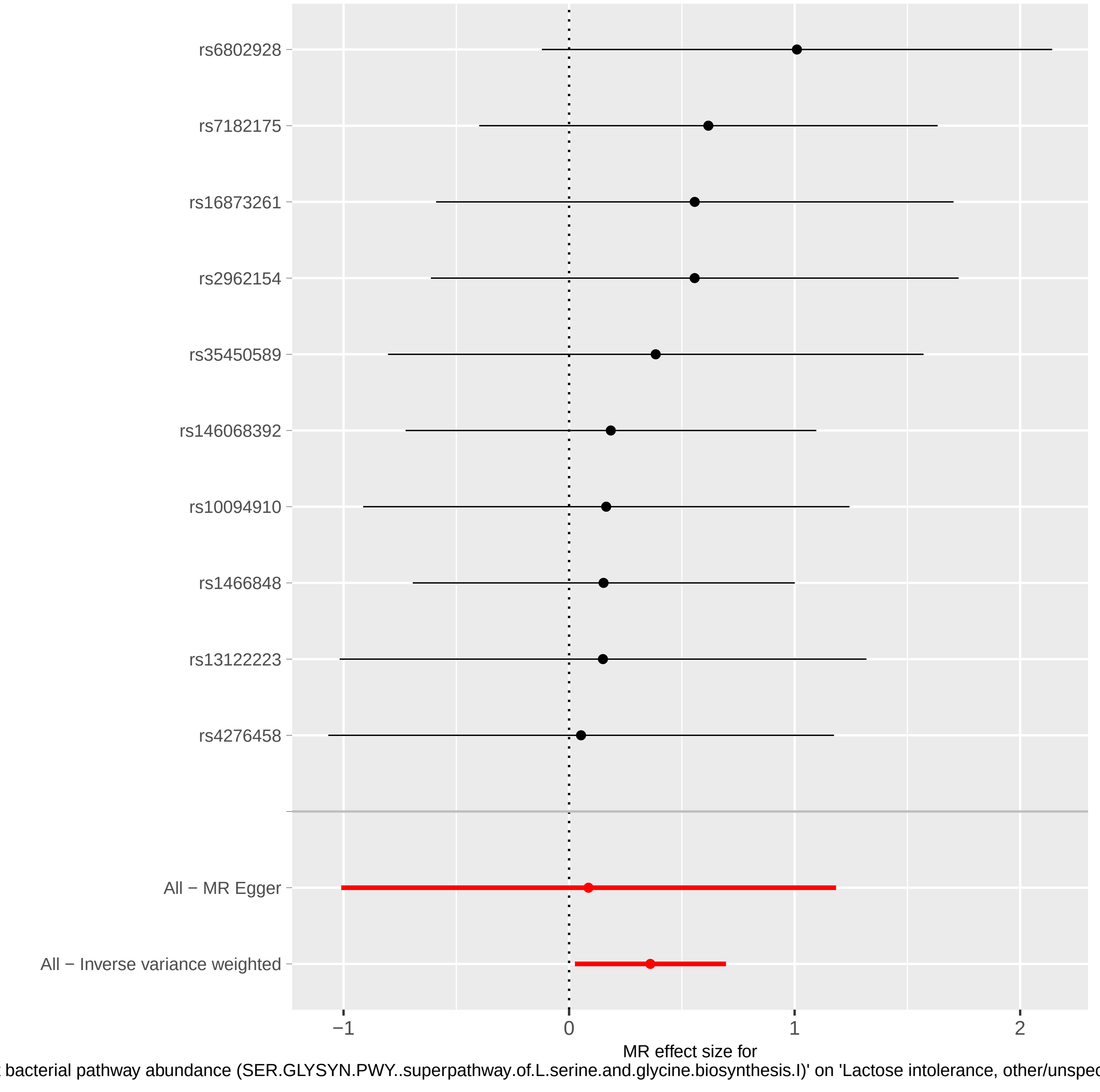

rs6802928
rs7182175
rs16873261
rs2962154
rs35450589
rs146068392
rs10094910
rs1466848
rs13122223
rs4276458
All − MR Egger
All − Inverse variance weighted
−1
0
1
2
MR effect size for
bacterial pathway abundance (SER.GLYSYN.PWY..superpathway.of.L.serine.and.glycine.biosynthesis.I)' on 'Lactose intolerance, other/unspec
